# Short-Chain Fatty Acids Elicit Differential Expression of Growth Factors and Pro-Inflammatory Cytokines in Immortalized Rat Enteric Glial Cells

**DOI:** 10.3390/nu18030436

**Published:** 2026-01-29

**Authors:** Michelle M. Beltran, Danielle M. Defries

**Affiliations:** 1 Department of Biology, University of Winnipeg, Winnipeg, MB R3B 2E9, Canada; 2 Department of Kinesiology and Applied Health, University of Winnipeg, Winnipeg, MB R3B 2E9, Canada

**Keywords:** enteric glial cells, short-chain fatty acids, growth factors, butyrate, propionate, transforming growth factor β-1, glial cell line-derived neurotrophic factor, enteric nervous system, pro-inflammatory cytokines

## Abstract

**Background/Objectives**: Enteric glial cells (EGCs) are non-neuronal cells of the enteric nervous system that contribute to intestinal homeostasis through interactions with the intestinal epithelium, enteric neurons, and resident intestinal immune cells. The objective of the current study was to determine how exposure of EGCs to short-chain fatty acids (SCFAs) would affect the expression of growth factors and pro-inflammatory cytokines, products of EGCs with known effects on intestinal epithelial barrier integrity. **Methods**: An enteroglial cell line was treated with low- (1 mM) or high- (10 mM) dose sodium butyrate or sodium propionate for 8 to 24 h, after which mRNA and protein levels of glial cell line-derived neurotrophic factor (GDNF), transforming growth factor β-1 (TGFβ-1), tumor necrosis factor α (TNFα), interleukin-6 (IL-6), and interleukin-1β (IL-1β) were measured using quantitative polymerase chain reaction assays and Western immunoblotting. **Results**: Only butyrate treatment for 8 and 24 h was associated with modest changes in GDNF mRNA. Neither SCFA elicited changes in TGFβ-1 mRNA. Despite this, high-dose butyrate and propionate were associated with reduced basal levels of TGFβ-1 protein as early as 12 h after treatment. Only butyrate was associated with a significant reduction in basal TNFα expression, which was present up to 24 h post-treatment. However, both butyrate (low- and high-dose) and propionate (high-dose only) elicited marked increases in IL-6 expression at all time points examined. Changes in cytokine mRNA levels were not mirrored at the protein level. **Conclusions**: SCFAs directly influence growth factor and cytokine expression in EGCs, but the functional implications of these changes in expression within the complicated milieux of the intestinal environment remain to be explored.

## 1. Introduction

The gastrointestinal (GI) tract is a major defense organ of the body, with the intestinal epithelium physically separating luminal contents from underlying intestinal tissue and preventing unwanted movement of pathogens and toxins into circulation. Integrity of the intestinal epithelial layer—and the tight junction complexes that seal the paracellular spaces between the epithelial cells—is influenced by several intestinal residents, including the microbiota [1,2], immune cells [3], the enteric nervous system [4], and the intestinal epithelial cells themselves [5]. Chronic inflammatory conditions, such as those experienced during intestinal dysbiosis, can alter the homeostatic state of these resident cells and ultimately compromise epithelial tight junction complexes, enhance intestinal permeability, and allow large molecules, bacteria, and toxins to enter circulation. An increase in intestinal permeability can have pathological consequences for a variety of systemic tissues and organs [6], with metabolic endotoxemia—the movement of bacterial-produced lipopolysaccharides into systemic circulation—proposed as the initial trigger for low-grade inflammation in peripheral organs, which is observed in metabolic disorders such as obesity and type 2 diabetes [7,8]. Thus, loss of epithelial barrier integrity and increased intestinal permeability may be an early contributor to systemic inflammation, insulin resistance, and other metabolic disturbances characteristic of obesity and type 2 diabetes.

Enteric glial cells (EGCs) are non-neuronal cells of the ENS that facilitate normal intestinal function by contributing to the integrity of the intestinal epithelial barrier, maintaining immune homeostasis, and supporting enteric neuronal health and function. Specific to the intestinal epithelium, active EGCs secrete soluble growth factors and other mediators—such as glial-derived neurotrophic factor (GDNF), transforming growth factor-β1 (TGF-β1), and s-nitrosoglutathione (GSNO)—which promote epithelial cell maturation, enhance the stability of epithelial tight junctions, and improve epithelial barrier integrity [9,10,11,12]. EGCs also contribute to innate immune responses in the intestine through secretion of pro-inflammatory cytokines in response to bacteria or inflammatory stimuli [13,14]. While cytokine secretion by active EGCs is considered homeostatic, chronic exposure of EGCs to pathogenic bacteria, endotoxins, or inflammatory mediators promotes the adoption of a reactive phenotype characterized by excessive cytokine production, which can exacerbate intestinal inflammation [15].

Short-chain fatty acids (SCFAs) are organic monocarboxylic acids produced via bacterial fermentation of non-digestible dietary carbohydrates, and to a lesser extent dietary protein, in the intestine [16]. Butyrate (four-carbon), propionate (three-carbon), and acetate (two-carbon) have been shown to attenuate intestinal inflammation and reduce intestinal permeability by altering cytokine and/or growth factor production secondary to G-protein-coupled receptor signaling and inhibition of histone deacetylases (HDACs), effects which are present in several cell types [17,18,19,20,21]. In both humans and mice, butyrate exerts immunomodulatory effects through downregulation of pro-inflammatory cytokines, such as TNFα, IL-6, and IL-1β, and upregulation of anti-inflammatory cytokines, such as IL-10 and TGF-β [22,23]. The exact nature of changes in cytokine production elicited by SCFAs is cell- and context-dependent, as SCFAs have also been shown to enhance IL-1β secretion through inflammasome activation in certain cell types [24]. In astrocytes of the central nervous system, butyrate inhibits production of LPS-induced cytokines, suggesting butyrate can exert anti-inflammatory effects in glia in the presence of chronic low-grade inflammation [25]. Likewise, SCFAs have also been shown to upregulate growth factor production in astrocytes as well as intestinal epithelial cells, presumably through a mechanism involving HDAC inhibition [26,27]. To date, no studies have examined how SCFAs may affect such factors in EGCs.

Therefore, the purpose of this study was to characterize the direct response of EGCs to SCFAs—with known potent HDAC inhibitory actions—on the production of mediators that have the potential to influence epithelial integrity and, thus, intestinal permeability in both homeostatic and inflammatory conditions.

## 2. Materials and Methods

### 2.1. Cell Culture

The EGCPK060399egfr enteroglial cell line (ATCC, Manassas, VA, USA; catalog #2690) was cultured in DMEM (Cytivia, Marlborough, MA, USA; catalog #AG29820908) supplemented with 10% fetal bovine serum (Gibco, Waltham, MA, USA; catalog #12483-020) and maintained at 37 °C with 5% CO_2_. When 80% confluent, cells were lifted and seeded into 12-well culture plates for Western blot experiments (seeding density of 4.0 × 10^4^ cells/well) or 6-well culture plates for qPCR experiments (seeding density of 1 × 10^6^ cells/well). Cells were grown to 80% confluence before exposure to experimental treatments. Cells between passages 4 and 15 were used for all experiments [28].

### 2.2. Treatment Conditions

To assess HDAC inhibition by SCFAs, ECGs were treated with 0.001 mM, 0.01 mM, 0.1 mM, 1 mM, and 10 mM sodium butyrate (MilliporeSigma, Burlington, MA, USA; catalog #303410) or sodium propionate (MilliporeSigma, catalog #P1880) for 2 h. Based on higher acetylation of histone H3, an indication of HDAC inhibitory activity, 1 mM and 10 mM were chosen as the doses of SCFAs to use in further experiments. For analysis of cytokine production, cells were treated with 1 mM and 10 mM sodium butyrate or 1 mM and 10 mM sodium propionate, with or without 10 ng/mL IFN–gamma (Abcam, Cambridge, UK; catalog #280340), for 8, 12, or 24 h (qPCR experiments) or 18 or 24 h (Western blotting experiments). For analysis of growth factor production, cells were treated with 1 mM or 10 mM sodium butyrate or sodium propionate for 8, 12, or 24 h (qPCR experiments) or for 8, 12, 18, or 24 h (Western blotting experiments). Doses of butyrate and propionate were chosen based on Western blotting results demonstrating the highest levels of acetylated histone H2 and H3, indicating HDAC inhibition, with 1 mM and 10 mM (Supplementary Material Figure S1). Treatment times were selected based on results from preliminary experiments (available at https://doi.org/10.5683/SP3/ZANOIJ (accessed on 15 December 2025).

### 2.3. Cell Viability Assays

The viability of EGCs treated with SCFAs was assessed using the Cell Counting Kit-8 (CCK-8) assay (Abcam, catalog #228554), following the manufacturer’s protocol. EGCs (1.0 × 10^4^/well) were seeded onto 96-well plates and grown to 80% confluence prior to exposure to treatments (*n* = 4 independent wells/treatment). Cells were incubated with butyrate or propionate for 8, 12, 18, and 24 h before the addition of CCK-8 reagent for 1 h at 37 °C and 5% CO_2_. Incubation times were chosen based on the treatment times with compounds for major outcome experiments. Absorbance of the samples was measured at 450 nm using a BioTek Epoch-2 Microplate Reader (BioTek, Winooski, VT, USA; model #EPOCH2C). Cell viability was calculated by normalizing blank-adjusted absorbance values to the average of the control cells.

### 2.4. RNA Extraction and Reverse Transcription

After treatment, cells were pelleted and stored at −80 °C for 18–24 h. Total cellular RNA was extracted from pellets (1 extraction replicate per sample) using the RNAqueous^TM^-4PCR Kit (Invitrogen, Waltham, MA, USA; catalog #AM1914) according to the manufacturer’s instructions. RNA was eluted with two centrifugation steps, each with 35 µL elution buffer (pre-heated to 75 °C) for a total volume of 70 µL eluted RNA. RNA concentration and purity (A260:280 and A260:230 ratios) were determined using the Nanodrop One Micro-UV/Vis Spectrophotometer (Thermo Fisher Scientific, Waltham, MA, USA; catalog #ND-ONE-W). Samples with A260:A280 ratios of 1.8–2.0 were used for complementary DNA (cDNA) synthesis. If samples did not possess acceptable A260/280 and/or A260/230 ratios, RNA was purified using the RNA Cleanup and Concentrator 5 Kit (Zymo Research, Irvine, CA, USA; catalog #R1013) following the manufacturer’s instructions. RNA integrity was verified by resolving samples on a 1% TAE-agarose gel with 2x RNA loading dye (1:1, *v*/*v*) (Life Technologies, Waltham, MA, USA: R0641) stained with SYBR Safe DNA Gel Stain (Thermo Fisher Scientific, catalog #2687599) using a Sub-Cell GT Agarose Gel Electrophoresis System (Bio-Rad, Hercules, CA, USA; catalog #1704401). Gels were imaged using the Bio-Rad GelDoc Go Imaging System (catalog #12009077), with Image Lab Touch Software (version 6.1) used to check the integrity of the 28S and 18S RNA bands. A 28S:18S ratio of 2:1 and distinct bands with no smearing was considered indicative of intact RNA suitable for complementary (cDNA) synthesis. RNA samples were stored at −80 °C (maximum storage time of 48 h) prior to performing reverse transcription.

Overall, 1000 ng of total RNA was reverse-transcribed into complementary DNA (cDNA) using the High-Capacity cDNA Reverse Transcription kit (Thermo Fisher Scientific, catalog #4368814), which uses random primers for cDNA priming, according to the manufacturer’s instructions. Reverse transcription was performed using a T100™ Thermal Cycler (Bio-Rad) using the following specifications: 25 °C for 10 mins; 37 °C for 120 mins; 85 °C for 5 min; hold at 4 °C. A no-RT control was included in each experiment. cDNA was diluted to 1 ng/uL and aliquoted to working stocks, which were stored at −80 °C.

### 2.5. Quantitative Reverse Transcription–Polymerase Chain Reaction (qRT-PCR)

Primers were designed based on previously published sequences or using the National Centre for Biotechnology Information’s Primer BLAST tool (https://www.ncbi.nlm.nih.gov/tools/primer-blast/ (accessed on 31 August 2023)), retaining all default parameters except minimum PCR product size (changed to 90 bp) and maximum PCR product size (changed to 400 bp). Primers were selected based on the following parameters: primer length (20–25 nucleotides), GC content (40–60%), melting temperature (55–65 °C), hairpin temperature (below 40–45 °C), homodimer (ΔG less than 10–15%), heterodimer (ΔG less than 20%), no more than 2 replicates of the same bases, and similar melting temperatures of forward and reverse primers (≤3 °C difference). OligoAnalyzer (https://www.idtdna.com/pages/tools/oligoanalyzer (accessed on 31 August 2023)) was used to check primer hairpin temperature, homodimer percentage, and heterodimer percentage to ensure that chosen primers would not result in primer dimers. All primers were ordered from Eurofin Genomics (https://eurofinsgenomics.com (accessed on 7 September 2023)). Primer sequences and characteristics are presented in Tables S1 and S2.

Optimal annealing temperature for each primer was determined by performing a temperature gradient analysis using the Veriflex temperature control function in the Quant Studio 5 Real-Time PCR System (Thermo Fisher Scientific, catalog #4385612), testing annealing temperatures from 50 °C to 70 °C. In addition, a melt curve analysis was used to determine primer specificity. A 9-point standard curve (3.91 ng/µL–1000 ng/µL) was used to determine the amount of cDNA template for further PCR assays and primer efficiency. Efficiencies of 90% to 110% were accepted. Experimental samples were assayed in triplicate, with an inter-plate control sample included on all plates to control for variability between plates (and runs). Thermocycling parameters were as follows: 1 cycle at 95 °C for 2 min; 40 cycles of denaturation at 95 °C for 5 s; and annealing using the primer-specific annealing temperature for 30 s. All qRT-PCR assays were performed in a Quant Studio™ 5 PCR system (Thermo Fisher Scientific, 96-well and 0.2 mL block) using Fast SYBR™ Green Master Mix (Thermo Fisher Scientific, catalog #4385612), with a 10 µL reaction volume for all samples.

Results from qPCR were analyzed using relative quantification with the 2^−ΔΔCT^ method to assess changes in gene expression relative to a reference sample (control group) [29]. The stability and intra/intergroup variability of three reference genes (GAPDH, b-actin, and 18S) were analyzed using NormFinder, with a stability value less than 0.15 considered stable [30]. The geometric mean of the CT values of the two most stable reference genes was used to determine ΔCT (all targets were normalized against the geometric mean of the two reference genes).

### 2.6. SDS-PAGE and Western Immunoblotting

After treatment, cells were lysed in RIPA buffer (150 mM NaCl, 50 mM Tris·HCl pH 7.4, 0.1% SDS, 1% NP-40, 0.5% sodium deoxycholate) supplemented with 1× protease/phosphatase inhibitor (Thermo Fisher Scientific, catalog #78440) at room temperature for 10 min. The protein concentration of the lysate was quantified using the Pierce™ bicinchoninic acid (BCA) assay (Thermo Fisher Scientific, catalog #23225). Protein extracts (5 μg) were separated by SDS-PAGE using 1× Tris–glycine running buffer (25 mM Tris base, 192 mM glycine, 0.1% SDS) in a Mini-Protean Tetra Cell System (Bio-Rad, catalog #1658000). Protein transfer to PVDF membranes was achieved using the Trans-Blot Turbo Transfer System (Bio-Rad, catalog #1704150), and efficiency of transfer was assessed using the MemCode^TM^ Reversible Protein Stain Kit (ThermoFisher Scientific, catalog #24585).

Membranes were blocked in 3% BSA-TBST before being probed with primary antibodies (histone H2B, catalog #12364; acetyl-histone H2B, catalog #12799; histone H3, catalog #4499; acetyl-histone H3, catalog #9649, Cell Signaling Technology, Danvers, MA, USA; IL-1β, catalog #9722; IL-6, catalog #9324; GDNF, catalog #176564; TGF-β1, catalog #215715; TNFα, catalog #307164, Abcam, Cambridge, UK). All primary antibodies were diluted 1:1000 in 3% BSA-TBST. Membranes were then incubated with horseradish peroxidase-conjugated secondary antibodies (diluted 1:10,000 in 1% BSA-TBST for histone H3, IL-1β, GDNF, and TGF-β1; diluted 1:5000 in 1% BSA-TBST for all other antibodies). Immunoreactive bands were visualized by chemiluminescence (Clarity^TM^ Western ECL Substrate Kit, Bio-Rad, Hercules, CA, USA; catalog #170-5061). Relative intensity of the protein bands was quantified using the ChemiDoc^TM^ XRS imaging system (Bio-Rad, Hercules, CA, USA) and Image Lab^TM^ Software version 6.1 (Bio-Rad). Each target protein was normalized against abundant and consistent protein bands, as detected by MemCode^TM^ protein stain [31]. The fold change compared to control was calculated by dividing the normalized protein target by the normalized control sample [32,33].

### 2.7. Statistical Analysis

Cell treatment experiments were repeated on separate days, and data from these independent experiments were used to calculate group means. Statistical analysis was performed using GraphPad PRISM (Version 10.2.3) and SPSS Version 29.0.2.0 (IBM Corp., Armonk, NY, USA). Shapiro–Wilk and Levene’s tests were used to assess normality and homogeneity of variance of the data. If the data were not normally distributed or did not have homogeneity of variance, the data were transformed. Statistical differences between group means were tested using one-way ANOVA and Tukey’s multiple comparisons test. Outliers were identified using the Robust Regression and Outlier Removal (ROUT) method (Q = 1%) [34], and only one outlier was removed from groups. Results with a *p*-value of *p* ≤ 0.05 were considered statistically significant.

## 3. Results

**Figure 1:**
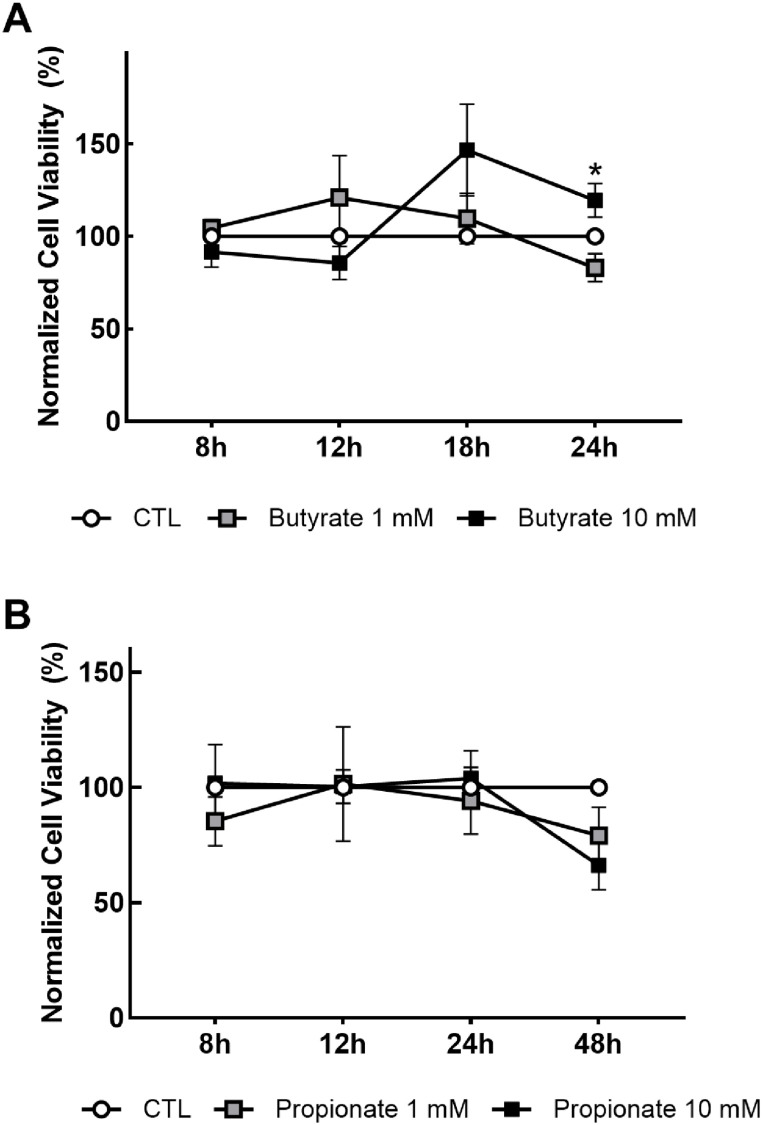
Cell viability in enteric glial cells treated with short-chain fatty acids. EGCPK/060399egfr enteroglial cells were treated with sodium butyrate (NaB; (**A**)) or sodium propionate (NaP; (**B**)) for 8, 12, 18, and 24 h. A commercially available kit using a water-soluble tetrazolium dye was used to determine cell viability. Data are presented as means plus or minus SEM (*n* = 4 independent experiments). * denotes significantly different from butyrate 1 mM (*p* < 0.05).

Our assessment of growth factor and cytokine production in response to SCFAs in EGCs involved treating cells with a low- and high-dose of SCFAs for up to 24 h. To determine if the doses of SCFAs and treatment durations used for qPCR and Western blotting experiments in this study affected cell viability, we performed CCK-8 assays in EGCs treated under the same conditions used in downstream analysis of gene and protein expression. The treatment durations chosen were 8, 12, 18, and 24 h, and were based on results from preliminary experiments (data repository link). In butyrate-treated cells, there were no significant main effects of time or treatment observed; however, a significant interaction between treatment and time was found, with cells treated with 10 mM butyrate having significantly higher viability compared to cells treated with 1 mM butyrate at the 24 h time point (Figure 1A). No significant effects of treatment, time, or interactions between treatment and time on cell viability were observed in cells treated with propionate (Figure 1B).

**Figure 2:**
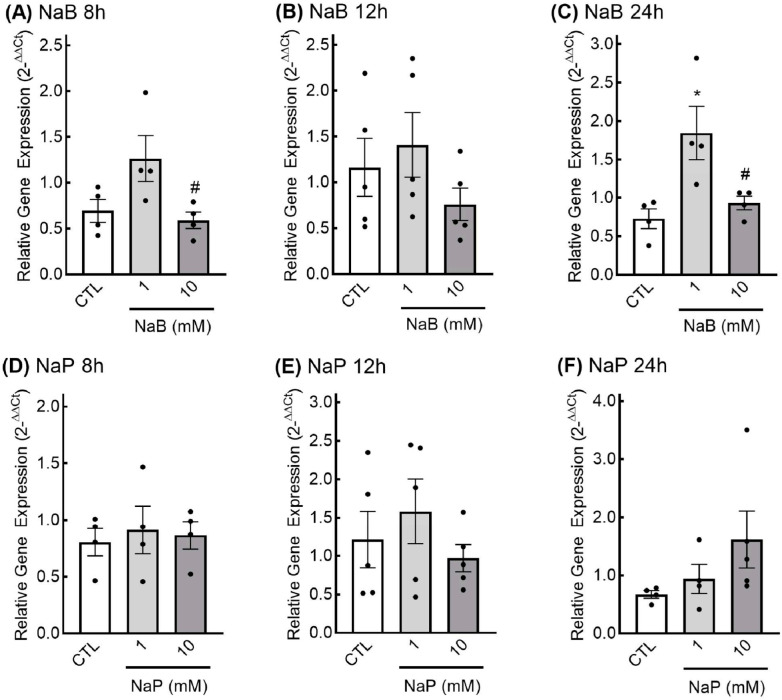
Glial-derived neurotrophic factor (GDNF) expression in response to short-chain fatty acid treatment in enteric glial cells. EGCPK/060399egfr enteroglial cells were treated with sodium butyrate (NaB) for 8, 12, and 24 h (**A**–**C**) or sodium propionate (NaP) for 8, 12, and 24 h (**D**–**F**). GDNF mRNA levels are expressed relative to untreated control (CTL), normalized to the geometric mean of the reference genes GAPDH and actin. Data are presented as means plus or minus SEM (*n* = 5 independent experiments). * denotes significantly different from CTL (*p* < 0.05); ^#^ denotes significantly different from 1 mM (*p* < 0.05).

***Effects of SCFAs on growth factor expression.*** Overall, SCFAs had modest influences on the expression of the growth factors examined in this study. Although GDNF expression appeared to be higher in cells treated with 1 mM butyrate, no significant differences in expression of GDNF were observed with butyrate treatment after 8 and 12 h (Figure 2A,B). However, after 24 h of treatment, GDNF expression was significantly higher in cells treated with 1 mM butyrate compared to both untreated cells and cells treated with 10 mM butyrate (Figure 2C). Propionate treatment did not induce significant changes in GDNF expression at any time point (Figure 2D–F).

**Figure 3:**
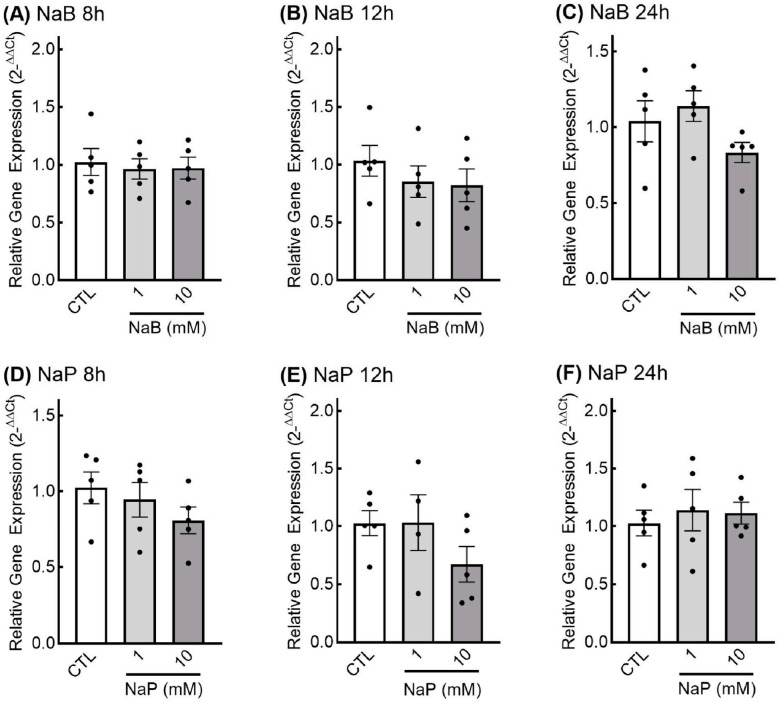
Relative expression of transforming growth factor β-1 (TGF β-1) in response to short-chain fatty acids in enteric glial cells. EGCPK/060399egfr enteroglial cells were treated with sodium butyrate (NaB) for 8, 12, and 24 h (**A**–**C**) or sodium propionate (NaP) for 8, 12, and 24 h (**D**–**F**). TGF β-1 mRNA levels are expressed relative to untreated control (CTL), normalized to the geometric mean of the reference genes GAPDH and actin. Data are presented as means plus or minus SEM (*n* = 5 independent experiments).

Similar results were observed regarding SCFAs and TGF-β1 expression. At the 8 h time point, no significant effects of butyrate on TGF-β1 expression were observed (Figure 3A). Although expression of TGF-β1 was lower compared to control in cells treated with 1 mM and 10 mM butyrate at the 12 h time point, differences were not statistically significant (Figure 3B). Similar effects were observed at the 24 h time point in response to 10 mM butyrate, but differences were not statistically significant (Figure 3C). TGF-β1 expression was reduced in cells treated with 10 mM propionate at both the 8 h and 12 h time points, but these differences were not statistically significant (Figure 3D,E). At the 24 h time point, no statistical differences between groups were observed (Figure 3F).

**Figure 4:**
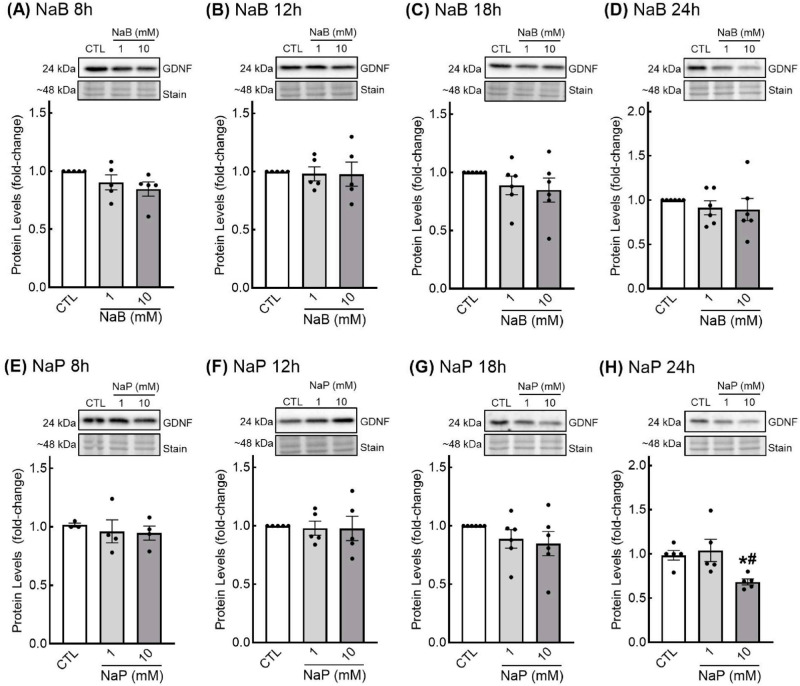
Glial cell line-derived neurotrophic factor (GDNF) protein levels in response to short-chain fatty acids in enteric glial cells. EGCPK/060399egfr enteroglial cells were treated with sodium butyrate (NaB) for 8, 12, 18, and 24 h (**A**–**D**) or sodium propionate (NaP) for 8, 12, 18, and 24 h (**E**–**H**). Protein levels were normalized to total protein levels and expressed as fold change relative to untreated cells (CTL). Representative images of GDNF blots and total protein stain are presented. Data are means plus or minus SEM (*n* = 6 independent experiments). * denotes significantly different from CTL (*p* < 0.05); ^#^ denotes significantly different from 1 mM (*p* < 0.05).

***Effects of SCFAs on growth factor protein levels.*** Since proteins are the final effectors of a gene’s action, we next examined the effect of SCFAs on the protein levels of GDNF and TGF-β1. For butyrate and GDNF, higher levels of GDNF mRNA did not result in higher levels of GDNF protein. In fact, there appeared to be a slight reduction in GDNF protein levels with both low- and high-dose butyrate and propionate treatment at the 18- and 24-h treatment time points (Figure 4C,D,G,H). However, only treatment with 10 mM propionate for 24 h resulted in significantly lower GDNF protein levels in EGCs (Figure 4H). No differences in GDNF protein levels were observed with butyrate or propionate treatment after 8 h or 12 h (Figure 4A,B,E,F).

**Figure 5:**
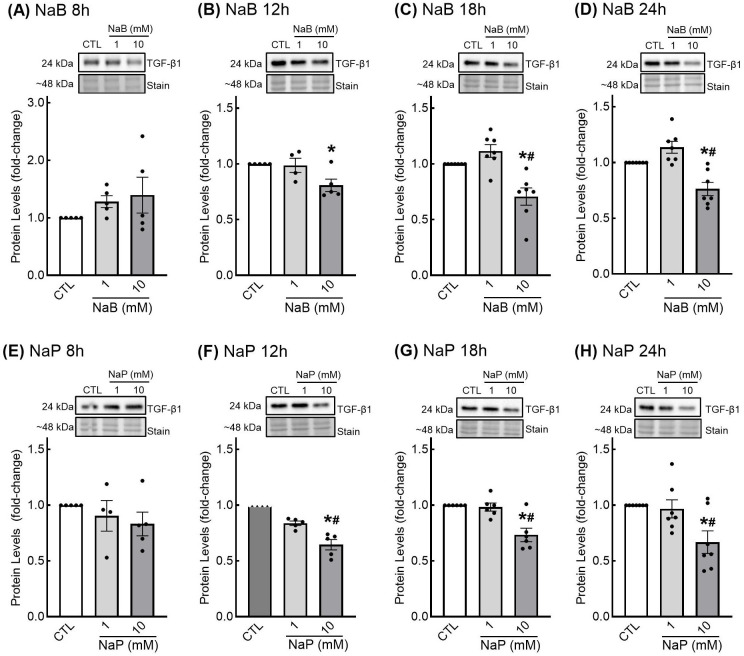
Transforming growth factor-β1 protein levels in response to short-chain fatty acids in enteric glial cells. EGCPK/060399egfr enteroglial cells were treated with sodium butyrate (NaB) for 8, 12, 18, and 24 h (**A**–**D**) or sodium propionate (NaP) for 8, 12, 18, and 24 h (**E**–**H**). Protein levels were normalized to total protein levels and expressed as fold change relative to untreated cells (CTL). Representative images of TGF-β1 blots and total protein stain are presented. Data are means plus or minus SEM (*n* = 6 independent experiments). * denotes significantly different from CTL (*p* < 0.05); ^#^ denotes significantly different from 1 mM (*p* < 0.05).

The effects of SCFAs on TGF-β1 protein levels are presented in Figure 5. There were no significant differences in TGF-β1 protein in response to either 1 mM butyrate or 1 mM propionate at any of the time points tested; however, 10 mM butyrate and 10 mM propionate treatment resulted in significantly lower levels of TGF-β1 protein after 12, 18, and 24 h of treatment (Figure 5B–D,F–H). This was not observed when cells were treated for 8 h (Figure 5A,E).

**Figure 6:**
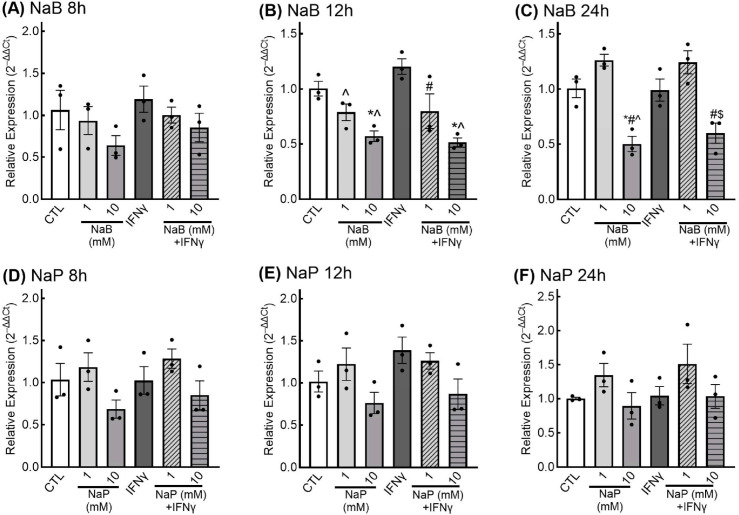
Relative expression of tumor necrosis factor α (TNFα) in response to short-chain fatty acids in enteric glial cells. EGCPK/060399egfr enteroglial cells were treated with sodium butyrate (NaB) for 8, 12, and 24 h (**A**–**C**) or sodium propionate (NaP) for 8, 12, and 24 h (**D**–**F**). TNFα mRNA levels are expressed relative to untreated control (CTL), normalized to the geometric mean of the reference genes GAPDH and actin. * denotes significantly different from CTL (*p* < 0.05); ^#^ denotes significantly different from 1 mM (*p* < 0.05); ^ denotes significantly different from IFNγ (*p* < 0.05); ^$^ denotes significantly different from 1mM + IFNγ (*p* < 0.05). Data are presented as means plus or minus SEM (*n* = 3 independent experiments).

***Effects of SCFAs on cytokine production in EGCs.*** TNFα, IL-6, and IL-1β are three pro-inflammatory cytokines produced and secreted by EGCs for which there is evidence to support a mechanism for control of expression by HDAC inhibitors [35]; therefore, we sought to determine if SCFAs could alter the expression of these three cytokines in EGCs. We examined the effects of low- and high-doses of butyrate and propionate in the presence and absence of INFγ, a potent inflammatory stimulus in glia [36], to separate the effects of SCFAs on cytokine production in basal and inflammatory states [37]. Despite attempts with several primer sets either designed, based on the published literature, or commercially available pre-designed and validated (TaqMan^®^ Gene Expression Assays and KiCqStart^®^ primers), we were unable to detect a PCR product for IL-1β; therefore, only results for TNFα and IL-6 expression are presented. Results for expression of TNFα are presented in Figure 6. In our experiments, IFNγ treatment did not result in an increase in expression of TNFα at any of the time points tested. Although there appeared to be a dose-dependent reduction in TNFα expression with butyrate—with or without IFNγ—as early as 8 h, reductions were not statistically significant (Figure 6A). After 12 h, cells treated with 1 mM butyrate retained small, non-significant reductions in TNFα, and cells treated with 10 mM butyrate displayed a significant reduction in TNFα expression (Figure 6B,C). Although 10 mM propionate treatment appeared to reduce TNFα expression at all three treatment time points, reductions did not reach statistical significance (Figure 6D–F).

**Figure 7:**
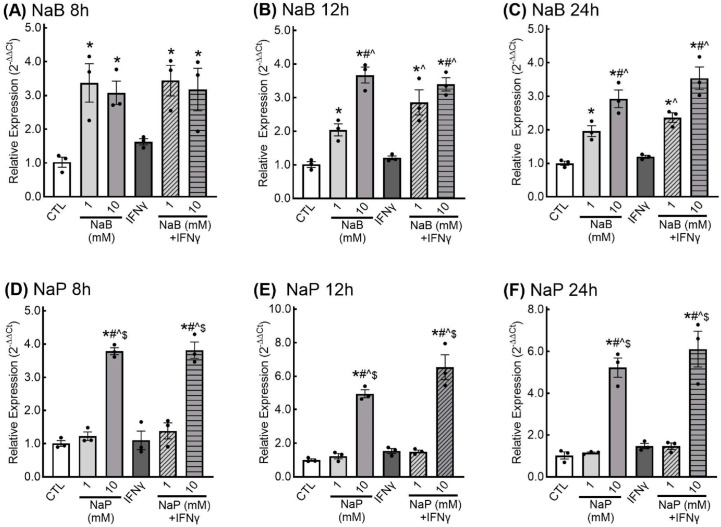
Relative expression of interleukin-6 (IL-6) in response to short-chain fatty acids in enteric glial cells. EGCPK/060399egfr enteroglial cells were treated with sodium butyrate (NaB) for 8, 12, and 24 h (**A**–**C**) or sodium propionate (NaP) for 8, 12, and 24 h (**D**–**F**). IL-6 mRNA levels are expressed relative to untreated control (CTL), normalized to the geometric mean of the reference genes GAPDH and actin. * denotes significantly different from CTL (*p* < 0.05); ^#^ denotes significantly different from 1 mM (*p* < 0.05); ^ denotes significantly different from IFNγ (*p* < 0.05); ^$^ denotes significantly different from 1mM + IFNγ (*p* < 0.05). Data are presented as means plus or minus SEM (*n* = 3 independent experiments).

For IL-6 expression, cells treated with both 1 mM and 10 mM butyrate had significantly higher relative expression of IL-6 at all three time points examined (Figure 7A–C). Although this appeared to be a dose-dependent effect at the 12- and 24-h treatment points, the differences in IL-6 relative expression between 1 mM and 10 mM butyrate were not statistically significant.

With propionate treatment, only cells treated with 10 mM propionate exhibited significantly higher IL-6 relative expression; this effect was observed at all three time points examined (Figure 7D–F).

**Figure 8:**
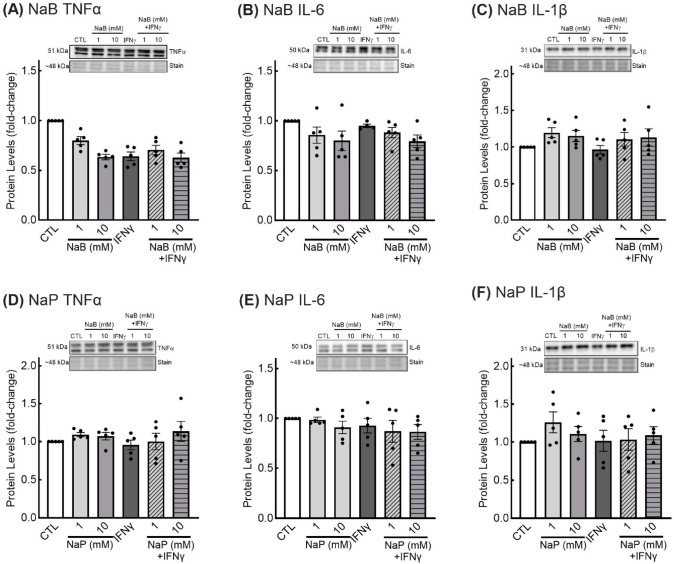
Protein levels of TNFα, IL-6, and IL-1β in response to short-chain fatty acids in enteric glial cells. EGCPK/060399egfr enteroglial cells were treated with sodium butyrate (NaB) (**A**–**C**) or sodium propionate (**D**–**F**) for 18 h (TNFα and IL-6) or 24 h (IL-1β), after which protein levels were assessed using Western immunoblotting. TNFα, IL-6, and IL-1β levels were normalized to total protein levels and expressed as fold change relative to untreated cells (CTL). Representative images of blots and total protein stain are presented. Data are means plus or minus SEM (*n* = 6 independent experiments).

Again, we examined protein levels of cytokines in EGCs treated with SCFAs to determine if changes at the transcript level were mirrored at the protein level. As we were able to find an antibody for IL-1β that produced clear and specific bands, we are able to report the effects of SCFAs on the protein levels of this cytokine. Despite the changes in TNFα and IL-6 that were observed at the gene level in response to SCFAs, there were no differences in TNFα, and IL-6 protein levels in cells treated with butyrate or propionate (Figure 8A,B,D,E). Similarly, there were no differences in protein levels of IL-1β in response to any of the treatments (Figure 8C,F).

## 4. Discussion

Integrity of the epithelial barrier is critical for the maintenance of intestinal homeostasis and prevention of systemic inflammation that may serve as an initial triggering factor underlying the development of metabolic disease [7,8]. EGCs play a pivotal role in maintaining intestinal homeostasis, in part through interactions with the epithelium that reinforce the strength of the intestinal epithelial barrier [12]. ECG activation and phenotype are influenced by intestinal conditions, and secretion of growth factors and cytokines by these cells can be differentially modified in ways that either enhance barrier integrity or compromise it [38]. The current study sought to determine if SCFAs—products of intestinal fermentation of indigestible carbohydrates—modify the production of growth factors and cytokines by EGCs in a manner that would promote barrier integrity, using doses reflective of what is found in the proximal colon [39]. Butyrate and propionate are established HDAC inhibitors, and HDAC inhibition has been linked to altered production of both growth factors and cytokines [17,18,19,20,21]. We tested the potential for HDAC inhibition of butyrate and propionate in our EGCs by assessing the level of acetylated histone H3 as a surrogate indicator. While butyrate was associated with a dose-dependent increase in acetylated histone H3, the results for propionate were not as convincing, and although there appeared to be an increase with high-dose propionate, this was not significant. This may relate to the reported lower HDAC inhibitor potency of propionate or the small sample size employed for this experiment. Although we are unable to definitively conclude that propionate exhibited HDAC inhibition in the EGC cells at this time, our results suggest that, while SCFAs may directly influence gene expression of cytokines in EGCs, protein levels are not affected. However, SCFAs modulate growth factor protein levels in EGCs. Despite the fact that EGCs are not in direct contact with luminal SCFAs produced by microbial fermentation, investigating the direct actions of SCFAs on EGCs is nonetheless relevant, as basolateral transport of SCFAs to underlying intestinal tissue facilitates the direct interaction of SCFAs with EGCs and other cells of the underlying GI tract layers [40].

GDNF is a growth factor secreted by EGCs that strengthens epithelial integrity by positively modulating levels and the arrangement of ZO-1 and occludin, tight junction proteins that seal the space between neighboring epithelial cells [9,41]. In addition to barrier-enhancing effects, GDNF secreted by glia enhances enteric neuronal branching and increases synapse number and size of synaptic clusters [42]. Positive regulation of GDNF by SCFAs has previously been shown in glia of the central nervous system. In astrocytes, SCFAs increase GDNF transcription via HDAC inhibition and hyperacetylation of the GDNF promoter [26]. In the current study, although low doses of butyrate were associated with small increases in GDNF mRNA, these results were not statistically significant. Furthermore, both butyrate and propionate were associated with slight reductions in GDNF protein.

Contrary to reports from previous studies in IECs [27,43], we observed significantly lower TGF-β1 protein levels when EGCs were treated with SCFAs, an effect which was most pronounced with butyrate, and not due to a significant loss of cell viability. To the best of our knowledge, the current study is the first to examine SCFA regulation of TGF-β1 production in enteric glia. TGF-β1 produced by both IECs and EGCs regulates IEC proliferation and enhances intestinal barrier integrity [44]. Our results showing reduced TGF-β1 suggest that SCFAs do not support intestinal barrier integrity through effects on TGF-β1 production by EGCs; however, the reduction in EGC-derived TGF-β1 may benefit intestinal homeostasis through other mechanisms. Several EGC subgroups have been identified based on the morphology and position within the layers of GI wall, and further classification based on distinct transcriptional profiles highlights unique functional attributes of EGC subgroups [45]. These functionally distinct EGCs may possess distinct responses to SCFAs that are consistent with their location and consequent functional role in supporting intestinal homeostasis. The EGC cell line used in the current study was derived from the jejunal myenteric plexus, and, given the pro-fibrotic nature of TGF-β1 [46], reduced TGF-β1 production may be a protective response by the subgroup of EGCs located close to GI muscle to reduce myofibroblast proliferation and prevent fibrosis. Indeed, the lower TGF-β1 production after butyrate treatment that has been observed in renal epithelial cells is considered favorable, given the established role of TGF-β1 in fibrotic renal disease [47]. Lower TGF-β1 production by EGCs exposed to SCFAs may contribute to the therapeutic potential for SCFAs in alleviating fibrosis in inflammatory bowel disease [48,49], although this requires confirmation in future studies.

As we observed no effect of SCFAs on TGF-β1 mRNA levels, changes in TGF-β1 protein could not be attributed to effects of HDAC inhibition on TGF-β1 transcription. Furthermore, if the HDAC inhibitory properties of SCFAs directly affected TGF-β1 expression, the expected effect would be hyperacetylation of the TGF-β1 promoter and increased expression of TGF-β1. Our results of lower TGF-β1 protein in the absence of changes at the transcriptional level point to post-transcriptional regulation of TGF-β1 by SCFAs in EGCs. In addition to their established effects on promoter-driven gene expression, HDAC inhibitors can regulate mRNA post-transcriptionally via effects on micro RNAs. TGF-β1 mRNA is under post-transcriptional control of micro RNAs, with miR-744 confirmed as a regulator of TGF-β1 [44]. While no evidence exists to date that miR-744 is specifically induced by SCFAs, butyrate has been shown to differentially modulate expression of micro RNAs [50], with levels of several miRNAs being upregulated. In addition, mechanisms separate from HDAC inhibition that control TGF-β1 protein levels could exist, as previous work has discovered a link between extracellular signal-regulated kinase signaling and translational control of TGF-β1 [47,51].

In the current study, SCFAs had a paradoxical effect on expression of the pro-inflammatory cytokines that were examined. While butyrate (but not propionate) induced a dose-dependent reduction in TNFα expression, most pronounced after 12 h of treatment, both butyrate and propionate elicited marked increases in IL-6 expression. This discordant effect on pro-inflammatory cytokine expression is puzzling when considered in relation to the intestinal epithelial barrier, as downregulation of TNFα would benefit barrier integrity, while upregulation of IL-6 would be detrimental. However, much like TGF-β1, the differential effects of pro-inflammatory cytokine production by EGCs upon exposure to SCFAs may reflect the multifaceted role of EGCs in intestinal homeostasis. Information on the physiological role of EGC-produced IL-6 is sparse; however, if similar to the effects of IL-6 produced by enteric neurons or glia of the CNS, this could involve regulatory T-cell differentiation or the promotion of neuronal growth and protection [52,53,54]. IL-6 as a pro-inflammatory cytokine has been shown to be involved in the pathogenesis of inflammatory bowel disease [55]; however, increased IL-6 expression by EGCs that we observed in counter to the documented beneficial effects of SCFAs in inflammatory bowel disease [56]. It is important to note that, while we did observe a dramatic increase in IL-6 mRNA upon SCFA treatment, this did not lead to a corresponding increase in intracellular IL-6 protein. One limitation of our study is that we did not measure levels of secreted cytokines in cell culture medium, and if secreted quickly, upregulation of the protein may not be captured by measuring intracellular protein levels, especially at later time points. Information on the direct effects of SCFAs on cytokine production in isolated glia is scant, and, to the best of our knowledge, the current study is the first study to report cytokine production in response to SCFAs in EGCs. Collectively, studies examining other cell types indicate that the influence of SCFAs on cytokine production is cell- and context-specific and dose-dependent. In myotubes, SCFA treatment reduced the expression of TNFα and IL-6, with associated reductions in nuclear factor κB (NFκB) and signal transducer and activator of transcription 3 (STAT3) signaling [57]. However, SCFAs induced differential effects on cytokine production in 3T3-L1 adipocytes, with reduced IL-6 and IL-1β expression, but no effect on TNFα expression in these cells [58,59]. In both of the aforementioned studies, SCFAs reduced pro-inflammatory cytokine expression after cells had been stimulated with either LPS or palmitic acid, but there were no changes in cytokine expression in unstimulated cells, as in the current study. In macrophages, butyrate can stimulate production of pro-inflammatory or anti-inflammatory cytokines, depending on the concentration of butyrate to which cells are exposed [60]. Whether SCFAs promote or suppress inflammation is determined by the dose to which cells are exposed, which affects the mechanism through which SCFAs can exert effects. Although the doses of butyrate and propionate tested in this study are representative of what appears in the colonic lumen [39], EGCs may not be exposed to such high concentrations after utilization by epithelial cells, and it is possible the doses used are high enough to promote selected inflammatory pathways, as seen with increased IL-6 mRNA. The same doses of butyrate and propionate that raised IL-6 mRNA levels also reduced TNFα mRNA, so dose alone cannot explain this. In the current study, attempts to examine the effect of SCFAs on cytokine production in an inflammatory state were not successful, as pre-treatment of EGCs with IFNγ—previously shown to stimulate activation and inflammation in EGCs [37]—did not elicit increases in TNFα, IL-6, or IL-1β. A similar lack of inflammatory response was observed when cells were treated with lipopolysaccharide or a combination of IFNγ + lipopolysaccharide (data repository link). The reason for this lack of pro-inflammatory cytokine response after exposure to an inflammatory stimulus remains unknown; however, this EGC cell line was created using viral transformation of primary EGCs [61], and it is possible that the initial viral transformation may have created a heightened state of basal inflammation not further increased by inflammatory stimuli. Alternatively, FBS used in culture medium could have interfered with our study of cytokine production, as FBS alone can induce cytokine secretion in several cell types [62]. However, other groups have been successful in eliciting inflammatory cytokine responses in the same EGC cell line with exposure to IFNγ and/or lipopolysaccharide, and cells were not reported to have been cultured in serum-free medium in these studies [63,64]. It is also worth noting that, in the current study, butyrate and propionate treatment elicited marked increases in IL-6, suggesting the cells were capable of an inflammatory response.

## 5. Conclusions

In conclusion, EGCs respond directly to SCFAs with a pattern of growth factor and cytokine production that appears inconsistent with support of intestinal epithelial barrier integrity; however, this pattern could positively impact other aspects of intestinal function and contribute to maintenance of homeostasis. The observed changes in pro-inflammatory cytokine expression with SCFA treatment were not manifested at the functional protein level. While our study provides preliminary evidence of the direct effects of SCFAs on growth factor and cytokine production in EGCs, future studies should examine the outcome of SCFA-induced changes in EGC-derived growth factors and cytokines in the in vivo context of the multicellular intestinal environment and the diverse physiological mechanisms regulating intestinal homeostasis.

## Data Availability

The original data presented in this study are openly available in Borealis at https://doi.org/10.5683/SP3/ZANOIJ.

## Supplementary Materials

The following supporting information can be downloaded at: https://www.mdpi.com/article/10.3390/nu18030436/s1, Figure S1. Levels of acetylated histone H3 in enteric glial cells (EGCs) treated with butyrate and propionate for 2 h. Table S1: Primer sequences and annealing temperatures for growth factor, cytokine, and endogenous control genes use in RT-qPCR assays in the EGCPK060399egfr enteroglial cell line; Table S2: Primer parameters for RT-qPCR assays in the EGCPK060399egfr enteroglial cell line. References [65,66,67,68,69,70,71] are cited in the Supplementary Materials.
