# Supplementary material for: Short-Chain Fatty Acids Elicit Differential Expression of Growth Factors and Pro-Inflammatory Cytokines in Immortalized Rat Enteric Glial Cells
Source: Nutrients. 2026 Jan 29;18(3):436. doi: 10.3390/nu18030436 (PMC12899430; doi:10.3390/nu18030436)
Supplement: Supplementary file 1 [file nutrients-18-00436-s001.zip › nutrients-4078865-supplementary.pdf]

Table S1. Primer sequences and annealing temperatures used in RT-qPCR assays in the EGCPK060399egfr enteroglial cell line.

Gene	NCBI Accession No.	Primer Sequence (5'-3')	Annealing temperature (°C)	Sequence source
GAPDH	NM_017008.4	F: GAAGGTCGGTGTGAACGGAT<br>R: ACCAGCTTCCCATTCTCAGC	60	Designed
18S	NR_046237.3	F: CGGCTACCACATCCAAGGAA<br>R: ATTGGAGCTGGAATTACCGC	58.8	Osuna et al. (2012) [65]
ACTB	NM_031144	F: CAGGGTGTGATGGTGGGTATGG<br>R: AGTTGGTGACAATGCCGTGTTC	63.6	Kim et al. (2011) [66]
GDNF	NM_001401780.1	F: GCCGAGACAATGTACGACAA<br>R: CTGGAGCCAGGGTCAGATAC	63.6	Shen et al. (2010) [67]
TGFB1	NM_021578.2	F: TACCATGCCAACTTCTGTCTGGG<br>R: ATGTTGGACAACTGCTCCACCTTG	60	Araujo et al. (2016) [68]
TNFA	X66539.1	F: CTGTGCCTCAGCCTCTTCTC<br>R: ACTGATGAGAGGGAGCCCAT	55.5	Designed
IL6	NM_012589.2	F: TAGAGTCACAGAAGGAGTGG<br>R: GCCAGTTCTTCGTAGAGAAC	57.4	Pousset et al. (1996) [69]
*IL-1β (#1)**	NM_031512.2	F: AGCTTCAGGAAGGCAGTGTC<br>R: TCAGACAGCACGAGGCATTT	55.4	Designed
*IL-1β (#2)**	NM_031512.2	F: AGTGAGGAGAATGACCTGTTC<br>R: CGAGATGCTGCTGTGAGATT	51-61<br>(attempted)	Di Liddo et al. (2015) [70]
*IL-1β (#3)**	N/A	F: ATTCCGAGCCAAGAGAACATAG<br>R: AGGCCACAGGGATTTTGTCG	51	Li et al. (2023) [71]
IL-1β (#4)#*	N/A	F: TAAGCCAACAAGTGGTATTC<br>R: AGGTATAGATTCTTCCCCTTG	52	Pre-designed<br>(Millipore Sigma)
IL-1β (#5a)$*	NM_031512.2	N/A	60	Pre-designed<br>(TaqMan® Assay lot 1)
IL-1β (#5b)$*	NM_031512.2	N/A	60	Pre-designed<br>(TaqMan® Assay lot 2)

* multiple primer sets for IL-1β represent several unsuccessful attempts at reliably amplifying IL-1β mRNA.

\# Commercially-available pre-designed primers (KiqStart®, Millipore Sigma)

$ Commercially-available pre-designed primers (TaqMan®, ThermoFisher Scientific). 5a and 5b represent two different lots of the same assay.

(65) Osuna, M.; Sonobe, Y.; Itakura, E.; Devnath, S.; Kato, T.; Kato, Y.; Inoue, K. Differentiation capacity of native pituitary folliculostellate cells and brain astrocytes. *J Endocrinol* **2012**, *213* (3), 231-237. DOI: 10.1530/joe-12-0043 From NLM.

(66) Kim, I.; Yang, D.; Tang, X.; Carroll, J. L. Reference gene validation for qPCR in rat carotid body during postnatal development. *BMC Res Notes* **2011**, *4*, 440. DOI: 10.1186/1756-0500-4-440 From NLM.

(67) Shen, L. H.; Li, Y.; Chopp, M. Astrocytic endogenous glial cell derived neurotrophic factor production is enhanced by bone marrow stromal cell transplantation in the ischemic boundary zone after stroke in adult rats. *Glia* **2010**, *58* (9), 1074-1081. DOI: 10.1002/glia.20988 From NLM.

(68) Araujo, A. P.; Diniz, L. P.; Eller, C. M.; de Matos, B. G.; Martinez, R.; Gomes, F. C. Effects of Transforming Growth Factor Beta 1 in Cerebellar Development: Role in Synapse Formation. *Front Cell Neurosci* **2016**, *10*, 104. DOI: 10.3389/fncel.2016.00104 From NLM.

(69) Pousset, F.; Fournier, J.; Legoux, P.; Keane, P.; Shire, D.; Soubrie, P. Effect of serotonin on cytokine mRNA expression in rat hippocampal astrocytes. *Brain Res Mol Brain Res* **1996**, *38* (1), 54-62. DOI: 10.1016/0169-328x(95)00324-l From NLM.

(70) Di Liddo, R.; Bertalot, T.; Schuster, A.; Schrenk, S.; Tasso, A.; Zanusso, I.; Conconi, M. T.; Schäfer, K. H. Anti-inflammatory activity of Wnt signaling in enteric nervous system: in vitro preliminary evidences in rat primary cultures. *J Neuroinflammation* **2015**, *12*, 23. DOI: 10.1186/s12974-015-0248-1 From NLM.

(71) Li, D.; Li, X.; Zhang, J.; Tang, Z.; Tian, A. The immunomodulatory effect of IL-4 accelerates bone substitute material-mediated osteogenesis in aged rats via NLRP3 inflammasome inhibition. *Front Immunol* **2023**, *14*, 1121549. DOI: 10.3389/fimmu.2023.1121549 From NLM.

Table S2. Primer parameters for RT-qPCR assays in the EGCPK060399egfr enteroglial cell line.

Gene	cDNA amount (ng)	Amplicon Size (bp)	Forward Melting Temp (°C)	Reverse Melting Temp (°C)	Annealing Temp (°C)	Primer Efficiency (%)
GAPDH	10	192	62.4	62.4	60	90.8
18S	10	N/A	62.4	60.4	58.8	92.6
ACTB	10	115	66.4	62.7	63.6	91.6
GDNF	10	N/A	60.4	64.5	63.6	91.5
TGFB	10	N/A	64.6	64.6	60	93.3
TNFA	250	126	64.5	62.4	55.5	112
IL6	250	209	60.1	59.9	57.4	92.2
IL-1β (#1)	10, 100, 250	239	59.6	59.6	55.4	Undetermined
IL-1β (#2)	10, 100, 250	N/A	54	55	51-61 (attempted)	Undetermined
IL-1β (#3)	10, 100, 250	N/A	62	64.7	51	Undetermined
IL-1β (#4)	10, 100, 250	N/A	56.8	57.7	52	Undetermined
IL-1β (#5)	10, 100, 250	74	N/A	N/A	60	90-110*
IL-1β (#6)	25, 50, 100	74	N/A	N/A	60	90-110*

*inherent property of all TaqMan® primers

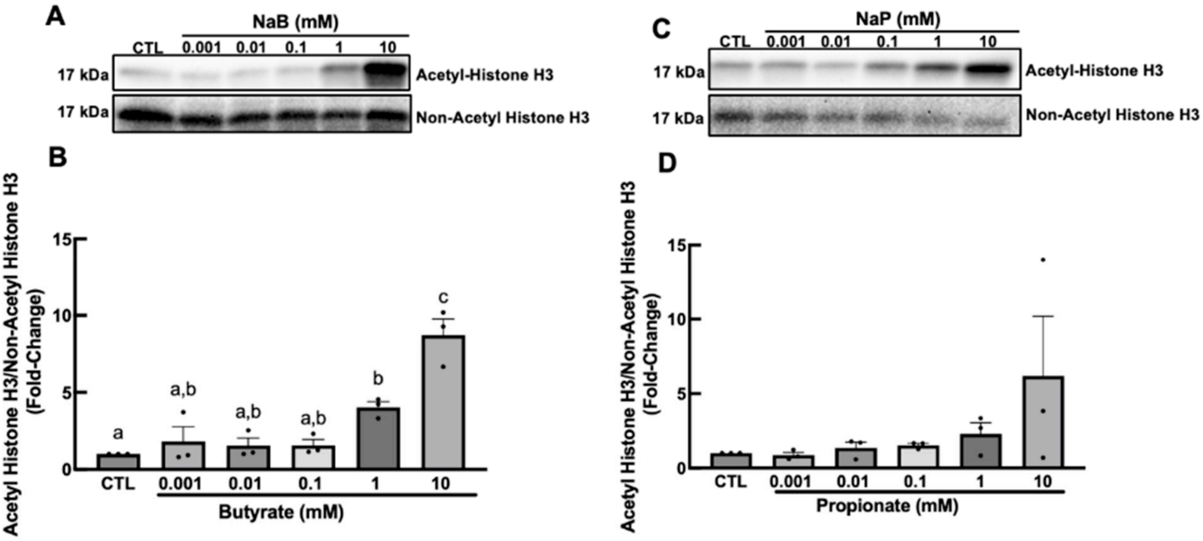

Figure S1. Levels of acetylated histone H3 in enteric glial cells (EGCs) treated with butyrate and propionate for 2 hours. (A-B) EGCs were treated with butyrate (0.001 mM, 0.01 mM, 0.1 mM, 1 mM and 10 mM) for 2 hours and (C-D) propionate (0.001 mM, 0.01 mM, 0.1 mM, 1 mM and 10 mM) for 2 hours. Levels of acetylated histone H3 were assessed using western blotting. Band intensities were quantified by densitometry and plotted as a ratio of acetylated histone H3 to non-acetylated histone H3. Data are expressed as means ± SEM (n=3 independent experiments). Means with different letters are significantly different
